# Metabarcoding Insights Into the Trophic Behavior and Identity of Intertidal Benthic Foraminifera

**DOI:** 10.3389/fmicb.2019.01169

**Published:** 2019-05-28

**Authors:** Panagiota-Myrsini Chronopoulou, Iines Salonen, Clare Bird, Gert-Jan Reichart, Karoliina A. Koho

**Affiliations:** ^1^Aquatic Biogeochemistry Research Unit, Ecosystems and Environment Research Programme, Faculty of Biological and Environmental Sciences, University of Helsinki, Helsinki, Finland; ^2^Biological and Environmental Sciences, University of Stirling, Stirling, United Kingdom; ^3^Department of Ocean Systems, NIOZ-Royal Netherlands Institute for Sea Research and Utrecht University, Den Burg, Netherlands

**Keywords:** metabarcoding, benthic foraminifera, trophic strategy, benthic food web, benthic microbial ecology, molecular phylogeny

## Abstract

Foraminifera are ubiquitous marine protists with an important role in the benthic carbon cycle. However, morphological observations often fail to resolve their exact taxonomic placement and there is a lack of field studies on their particular trophic preferences. Here, we propose the application of metabarcoding as a tool for the elucidation of the *in situ* feeding behavior of benthic foraminifera, while also allowing the correct taxonomic assignment of the feeder, using the V9 region of the 18S (small subunit; SSU) rRNA gene. Living foraminiferal specimens were collected from two intertidal mudflats of the Wadden Sea and DNA was extracted from foraminiferal individuals and from the surrounding sediments. Molecular analysis allowed us to confirm that our foraminiferal specimens belong to three genetic types: *Ammonia* sp. T6, *Elphidium* sp. S5 and *Haynesina* sp. S16. Foraminiferal intracellular eukaryote communities reflected to an extent those of the surrounding sediments but at different relative abundances. Unlike sediment eukaryote communities, which were largely determined by the sampling site, foraminiferal intracellular eukaryote communities were driven by foraminiferal species, followed by sediment depth. Our data suggests that *Ammonia* sp. T6 can predate on metazoan classes, whereas *Elphidium* sp. S5 and *Haynesina* sp. S16 are more likely to ingest diatoms. These observations, alongside the use of metabarcoding in similar ecological studies, significantly contribute to our overall understanding of the ecological roles of these protists in intertidal benthic environments and their position and function in the benthic food webs.

## Introduction

Benthic foraminifera are ubiquitous, single-celled protists. Due to their opportunistic character (e.g., Moodley et al., 2000; Woulds et al., 2007), foraminifera can take advantage of their environment very efficiently and they are able to thrive in a wide variety of marine environments. Their ecology is complex, with some species harboring photosynthetically active symbionts or kleptoplasts (e.g., Hallock, 2000; LeKieffre et al., 2018; Schmidt et al., 2018) and other various endobionts (e.g., Bernhard, 2003; Tsuchiya et al., 2015; Bernhard et al., 2018), of which some may be used in direct carbon transfer to host foraminifera (Tsuchiya et al., 2018). Foraminifera are generally considered as heterotrophic organisms with multiple feeding strategies. Of these, carnivory and predation are well-documented among planktonic foraminifera (Boltovskoy and Wright, 1976; Bé et al., 1977), however, for benthic foraminifera we rely only on experimental observations, which suggest that some species may pray on nematodes or other metazoans (Suhr et al., 2008; Dupuy et al., 2010). Instead, a number of experimental studies suggest that phototrophs provide an important source of organic carbon and nutrients to benthic foraminifera (Moodley et al., 2000; Nomaki et al., 2005, 2006; Larkin et al., 2014; Jeffreys et al., 2015; LeKieffre et al., 2017). Generally, however, there is a distinct lack of *in situ* evidence of species-specific feeding modes and ecological relationships among benthic foraminifera and sediment micro- and meiofauna due to the difficulties of studying these processes in nature. Understanding species-specific feeding behaviors is crucial to unraveling the adaptability strategies of benthic foraminifera in their habitats, understanding the benthic food webs structure and addressing implications for the global marine benthic biogeochemical cycles.

Metabarcoding may provide new insights into life strategies and *in situ* feeding modes of foraminifera and allow the identification of potential species-specific preferences. This approach has been successfully applied to investigate the microbiome and potential feeding preferences of marine eukaryotes, such as copepods (Ray et al., 2016) and nematodes (Schuelke et al., 2018). Recently, 16S rRNA metabarcoding was also used to study the intracellular bacterial composition of pelagic foraminifera to elucidate their ecological strategies (Bird et al., 2017, 2018). Cloning and shallow Sanger sequencing have been recently used to demonstrate the multiple diatom associations within an individual benthic foraminifer, suggesting that the host can shuffle its symbionts in response to thermal stress (Schmidt et al., 2018). However, the application of metabarcoding in benthic foraminifera is yet to be tested.

A good taxonomic resolution is essential in solving species-specific feeding preferences and potential niche and resource partitioning among foraminiferal population. For planktonic foraminifera, cryptic species have been shown to display niche differentiation within the water column (Weiner et al., 2012) as well as geographically on a spatial scale (Aurahs et al., 2009). Metabarcoding allows not only the identification of prey but also the cryptic diversity of the feeder that is not readily distinguished morphologically (e.g., Miller et al., 1982; Pawlowski and Holzmann, 2008; Schweizer et al., 2011; Pillet et al., 2012; Darling et al., 2016; Roberts et al., 2016; Lei et al., 2017). The 37f hypervariable region of the 18S (SSU) rRNA gene is commonly used in foraminiferal molecular studies (Pawlowski, 2000). As this helix region is foraminifera-specific and able to identify foraminifera to species level (Lecroq et al., 2011), it has been proposed as a DNA barcode (Pawlowski and Holzmann, 2014). Yet, the 37f region wider use in foraminiferal identification is impeded by the under-representation in public databases. In contrast, the V9 hypervariable region of the 18S rRNA gene is well-represented in public databases, and it captures a large eukaryotic diversity including that of protists (Amaral-Zettler et al., 2009; Behnke et al., 2011; Pawlowski et al., 2011). However, this hypervariable region has not yet been considered for the taxonomic placement of benthic foraminifera.

Here, for the first time, to the best of our knowledge, we target the V9 hypervariable region of the 18S rRNA gene within benthic foraminiferal cells. In addition, the foraminiferal intracellular eukaryote communities are compared to those of their surrounding sediments to gain insights into the relative distribution of foraminiferal food sources in the sediment. Moreover, the observed intracellular eukaryote diversity is linked to external factors (e.g., site, habitat depth in sediment, and total sedimentary organic carbon and nitrogen content), as parameters like organic carbon availability and sediment depth have been shown to be important in structuring the intertidal foraminifera community (e.g., Thibault De Chanvalon et al., 2015; Mojtahid et al., 2016). The overall aim of this study is to identify species-specific trophic preferences of benthic foraminifera, and, in parallel to unravel their taxonomic identity.

## Materials and Methods

### Site Description and Sampling

Two intertidal mudflat localities (Supplementary Figure 1) were sampled in November 2015 at the Dutch Wadden Sea: Mokbaai (M) characterized by relatively sandy sediment with the presence of polychaete worm burrows (>10 cm depth), and de Cocksdorp (C) characterized by non-burrowed clay/mud sediment.

One sediment core (10 cm internal diameter) per site was sampled manually by pushing a core tube into the sediment during low tide and processed as described in Koho et al., 2018; see detailed steps in Supplementary Figure 1C. In short, three sub-cores (50 ml truncated syringes) were taken from the main core. Two of the sub-cores were transferred in a nitrogen-filled glove bag and sliced with 1 cm intervals down to 10 cm depth. Porewater was removed, centrifuging the sediment, and the solid phase was frozen to –20°C and transferred to the University of Helsinki, where it was freeze-dried. Then, sedimentary organic carbon and total nitrogen was measured with a Leico TruSpec^®^ Micro, following homogenization and decalcification (1 M HCl). The third sub-core was also sliced at 1 cm intervals down to 10 cm sediment depth and used to obtain environmental DNA (eDNA; referred to as sediment DNA) samples and foraminiferal specimens. Each sediment slice was subsampled (ca. 1–1.5 g sediment) with a sterile plastic spatula, the subsample was immediately frozen in liquid nitrogen and kept stored in –20°C until eDNA extraction. The rest of the slice was sieved with filtered seawater through a 125 μm mesh and intact foraminiferal cells with visible protoplasm picked under a microscope (see Supplementary Table 1 for details on collected living specimens). Vitality was confirmed based on movement of foraminifera under oxygenated conditions (see Koho et al., 2011), and foraminifera specimens were identified to genus level morphologically. Subsequently, each living specimen was washed three times with sterile artificial seawater, transferred into RNA*later* solution (Invitrogen^TM^), which dissolves the calcite test, and stored at +4°C until further molecular analyses.

### DNA Extraction, Amplification, and Sequencing

DNA was extracted from foraminiferal individuals following the DOC (sodium deoxycholate) method (Holzmann and Pawlowski, 1996). Before placement in the DOC buffer, the naked foraminiferal cells were washed again 3–5 times in sterile artificial seawater (Red Sea’s Coral Pro Salt, salinity adjusted to 29‰), to clean the cells of any surficial organisms and eliminate RNA*later* traces (see Bird et al., 2017). The partial SSU rRNA gene (approximately 550 base pairs (bp)) of two specimens (M1C and M5B) was genotyped by conventional methods according to (Darling et al., 2016). Sediment DNA (ca. 0.25 g) was extracted using the PowerSoil^®^DNA Isolation Kit (MoBio, Carlsbad, CA, United States), according to the manufacturer’s instructions.

DNA from foraminifera and sediment samples was amplified alongside three extraction controls containing no template with either (i) DOC and artificial seawater (two replicates) and (ii) the buffers of MoBio PowerSoil^®^DNA Isolation Kit. In addition, non-template PCR controls of the first and second (indexing) PCR (see below) were sequenced.

The V9 region of the 18S rRNA gene was targeted with the 1389F/1510R primers described by Amaral-Zettler et al. (2009), and widely used in ecological studies for the investigation of eukaryotic diversity (e.g., de Vargas et al., 2015; Sawaya et al., 2019; Pitsch et al., 2019). Primers were modified at the 5′ end to include overhang sequences (Illumina adapters) for the downstream sequencing (forward overhang (37 bp): 5′-ATCTACACTCTTTCCCTACACGACGCTCTTCCGATCT-3′; reverse overhang (34 bp): 5′-GTGACTGGAGTTCAGACGTGTGCTCTTCCGATCT-3′). Amplification reactions were performed on an Applied Biosystems Veriti 96 Well Thermal Cycler, using the Phusion Mastermix (Thermo Fisher Scientific) and following the manufacturer’s protocol. PCR conditions for foraminiferal DNA were as follows: 98°C for 1 min, 25 cycles of 98°C for 10 s, 67°C for 15 s and 72°C for 15 s, 12 cycles of 98°C for 10 s, 72°C for 15 s and 72°C for 30 s, with a final elongation of 72°C for 1 min. PCR conditions for sediment DNA were the same, except for the annealing temperature (72°C) and cycle numbers (25–30 cycles). Duplicate PCRs were performed and pooled in equal volumes, to minimize the intra-sample variance and obtain enough amplicon volume for Illumina library preparations. Pooled samples, including negative controls, were quality-checked on 1.5% w/v agarose gels. Prior to sequencing, PCR products were purified and a second indexing PCR (P7 unique index attached) was performed followed by magnetic bead purification as described in Salava et al. (2017). In order to mitigate the possibility of cross-contamination due to mistagging (Esling et al., 2015), unique barcodes were selected for the indexing PCR using BARCOSEL (Somervuo et al., 2018). Samples were sequenced on the Illumina MiSeq platform of the Laboratory of DNA Sequencing and Genomics at the Institute of Biotechnology, Helsinki Institute of Life Science (HiLIFE).

### Processing of Sequences and Phylogenetic Analysis

Raw reads were de-multiplexed to samples based on their barcode sequences and MiSeq overhangs, primers, and barcode sequences were removed as described in Salava et al. (2017). Sequences were assembled to paired-end reads and quality-filtered in Mothur version 1.39.5 (Schloss et al., 2009). Minimum and maximum sequence lengths were set to 122 and 151 bp, respectively. No ambiguous sequences were allowed and the maximum number of homopolymers was set to 8. Quality-filtered reads were aligned against the SILVA database (release 128) and chimeric sequences were removed with the implementation of UCHIME algorithm (Edgar et al., 2011) in Mothur. Taxonomic assignment of all sequences was performed in Mothur against the SILVA database and taxonomic information was used in downstream clustering. Clustering into Operational Taxonomic Units (OTUs) was done using an arbitrary chosen 95% similarity sequence cutoff (e.g., Caron et al., 2009) in order to aggregate variation due to sequencing and PCR errors. Consensus taxonomy for each OTU was determined at 0.05 distance level. OTUs assigned to Foraminifera by SILVA were further compared to the PR^2^ (version 4.7) database (Guillou et al., 2013) to achieve genus level assignment. Representative sequences for each OTU were determined in Mothur as the centroids (sequence with the smallest distance to the other sequences) of the distance matrix created at the clustering stage. The representative sequences of OTUs that remained unclassified with the SILVA database, were aligned in a stand-alone BLAST search (Altschul et al., 1990) against the NCBI’s non-redundant nucleotide database. BLAST results were also used to confirm the identity of foraminiferal specimens at the genus level (Table 1).

**Table 1:** Foraminiferal specimens and their identity.

Specimen code	Depth (cm)	ID (PR^2^)	Genotype	Closest relative to most abundant OTU (BLAST)	BLAST ID (%)	No. of foram OTUs	% reads in most abundant OTU
M1B	0–1	*Amm*	NA	*A. aomoriensis* (GQ853573)	100	18	80.46
M1C^∗^	0–1	*Elph*	*Elph* sp. S5	*Elphidium* sp. S5 (KX962814)	100	23	99.52
M1D	0–1	*Amm*	NA	*A. aomoriensis* (GQ853573)	100	8	48.62
M2B	1–2	*Amm*	NA	*A. aomoriensis* (GQ853573)	100	21	81.37
M2E	1–2	NA	NA	NA	NA	7	80.39
M3A	2–3	*Amm*	NA	*A. aomoriensis* (GQ853573)	100	19	77.44
M3B	2–3	*Amm*	NA	*A. aomoriensis* (GQ853573)	100	19	78.39
M3D	2–3	*Hay*	*Hay* sp. S16	*Haynesina* sp. S16 (KX962996)	99	28	95.95
M4C	3–4	*Elph*	*Elph* sp. S5	*Elphidium* sp. S5 (KX962814)	100	17	98.95
M4D	3–4	NA	NA	NA	100	5	63.64
M5B^∗^	4–5	*Amm*	*Amm* sp. T6	*A. aomoriensis* (GQ853573)	100	19	94.73
M6A	5–6	*Amm*	NA	*A. aomoriensis* (KT989509)	100	7	84.77
M6B	5–6	*Elph*	*Elph* sp. S5	*Elphidium* sp. S5 (KX962814)	100	5	96.10
M7A	6–7	NA	NA	NA	NA	5	80.31
M7D	6–7	*Elph*	*Elph* sp. S5	*Elphidium* sp. S5 (KX962814)	100	26	99.52
M8A	7–8	*Amm*	NA	*A. aomoriensis* (GQ853573)	100	17	65.17
M8B	7–8	*Elph*	*Elph* sp. S5	*Elphidium* sp. S5 (KX962814)	100	27	78.10
M8D	7–8	*Elph*	*Elph* sp. S5	*Elphidium* sp. S5 (KX962814)	100	16	99.69
M9B	8–9	*Elph*	*Elph* sp. S5	*Elphidium* sp. S5 (KX962814)	100	22	99.68
M9F	8–9	*Amm*	NA	*A. aomoriensis* (GQ853573)	100	9	70.50
M10B	9–10	*Hay*	*Hay* sp. S16	*Haynesina* sp. S16 (KX962996)	99	25	89.90
M10C	9–10	*Elph*	*Elph* sp. S5	*Elphidium* sp. S5 (KX962814)	100	20	99.64
M10D	9–10	*Elph*	*Elph* sp. S5	*Elphidium* sp. S5 (KX962814)	100	20	99.75
C1A	0–1	*Elph*	*Elph* sp. S5	*Elphidium* sp. S5 (KX962814)	100	19	99.68
C1B	0–1	*Elph*	*Elph* sp. S5	*Elphidium* sp. S5 (KX962814)	100	24	99.65
C2D	1–2	*Elph*	*Elph* sp. S5	*Elphidium* sp. S5 (KX962814)	100	25	95.93
C3B	2–3	*Elph*	*Elph* sp. S5	*Elphidium* sp. S5 (KX962814)	100	24	84.88
C4C	3–4	*Elph*	*Elph* sp. S5	*Elphidium* sp. S5 (KX962814)	100	23	99.48


*^∗^For specimens M1C and M5B genotyping was conducted as described in Darling et al. (2016).*

*“M” indicates foraminiferal specimens from Mokbaai and “C” from de Cocksdorp. “Amm” stands for Ammonia sp., “Elph” for Elphidium sp. and “Hay” for Haynesina sp. NA = not applicable. Molecular identification at genus level was done via taxonomic assignment of the obtained foraminiferal OTUs based on the Protist Ribosomal Reference database (PR^*2*^) in Mothur. Samples M2E, M4D and M7A could not be assigned taxonomy at genus level, due to very low abundance of foraminiferal retrieved sequences from these specimens, thus their microscopic identification was used (Ammonia sp. for M2E and M7A; Elphidium sp. for M4D). Genetic types are as described in Darling et al. (2016), and here they refer to the closest relatives of the foraminiferal OTUs in each specimen based on a BLAST search. For comparison, the BLAST result for the most abundant foraminiferal OTU in each specimen is also given.*

OTUs with ≤ 8 and ≤ 10 sequence reads across the foraminiferal and sediment datasets, respectively, were removed. We set these thresholds empirically based on the cumulative sum of OTUs removed at increasing threshold in order to reduce the amount of rare diversity while preserving our sequencing effort (see Supplementary Figure 2). Filtering retained 99.86 and 99.03% of the total reads count for the foraminiferal and sediment dataset, respectively. Only two OTUs (unclassified Eukaryota) were excluded from the sediment dataset, as due to their abundance in the non-template PCR control (39,668 and 6,759 sequences, accounting for 84.15 and 14.34% of reads in the non-template PCR controls but only 0.46 and 0.37% of reads on average in the samples) they were considered contaminants in the PCR reactions. One more OTU was excluded because it was abundant in the kit extraction control (137 sequences, accounting for 29.40% of reads in the control but 0.00007% on average in the samples) indicating that it is a contaminant of the kit reagents. DOC extraction buffer controls returned low numbers of sequences (half the average number of sequences in the samples), which could either not be aligned to SILVA’s 18S database or were assigned to prokaryotes and thus filtered out by the Mothur pipeline with no interference to the downstream analysis.

In order to compare the diversity of eukaryotic communities found in foraminiferal hosts and in the surrounding sediment, OTUs belonging to phylum Retaria (called TF = Texel Foraminifera) were excluded from both datasets. Sediment OTUs are hereafter called “TS” (standing for Texel sediments) and intracellular foraminiferal eukaryote OTUs called “TIFC” (standing for Texel intracellular foraminiferal content).

Representative sequences of all the TFs and their closest relatives were aligned using the muscle algorithm (v3.8.31, Edgar, 2004) and edited in MEGA7 (Kumar et al., 2016). Maximum likelihood (ML) phylogenetic tree was constructed using MEGA7, after performing a “best model” analysis to select the best substitution model (Kimura 2-parameter model with discrete Gamma distribution rates among sites and assuming a certain fraction of sites (15.32%) to be evolutionarily invariable) according to BIC (Bayesian Information Criterion) (Hall, 2013). The tree was edited in Dendroscope (version 3.5.9; Huson et al., 2007) and Adobe Illustrator CC (2014 release).

### Statistical Analysis

Statistical analysis was done in R (version 3.4.2), using the packages phyloseq (version 1.22.3) (McMurdie and Holmes, 2013) and vegan (version 2.4-4) (Oksanen et al., 2015). DCA (detrended correspondence analysis) indicated that both the foraminiferal and sediment datasets are heterogeneous (length of first DCA axis > 4 standard deviations), thus unimodal models were applied for multivariate analysis. Available environmental data [sedimentary organic carbon and total nitrogen contents and their molar ratio (C/N)], sampling site and sample depth range (0–2, 2–6, and 6–10 cm) were considered as potential explanatory variables for the observed community variance. Automatic stepwise model building (ordistep in package vegan) was applied, in order to select the best fitting model based on the Akaike information criterion (AIC) and using permutation tests. Multicollinearity was checked by calculation of the variance inflation factors (VIFs) and only factors with VIF < 5 were considered.

### Accession Numbers

The DNA sequences representative of OTUs reported in this study were deposited in the Genbank database. A total of 65 foraminiferal sequences (TF) are under the accession numbers MK011309 – MK011373, 445 foraminiferal intracellular content sequences (TIFC) under the accession numbers MK012677 – MK013121, and 1,571 sediment sequences (TS) under the accession numbers MK020770 – MK022340. Moreover, the raw fastq files were deposited to SRA under the Sequence Read Archive (SRA) BioProject accession number PRJNA472012.

## Results

### Taxa (OTUs) Obtained and Sequencing Depth

DNA was analyzed from within 23 foraminiferal specimens from Mokbaai and 5 specimens from de Cocksdorp (Table 1). Additionally, sediment samples obtained from the same depths as foraminiferal specimens (0–10 cm for site M, 0–4 cm for site C) were used for metabarcoding along with the foraminifera.

A total of 2,847,274 sediment and 5,227,694 intracellular foraminiferal sequence reads were obtained, which after quality filtering were reduced to 1,881,013 for the sediment and 3,654,067 for the foraminiferal dataset. Chimera check removed another 0.56% of the sediment and 0.13% of the intracellular foraminiferal reads. The remaining reads were clustered into 6,949 OTUs for the sediment and 3,011 OTUs for the intracellular foraminiferal dataset. After filtering out OTUs with low number of reads (see Materials and Methods and Supplementary Figure 2) and non-eukaryote OTUs, 1,608 OTUs were obtained from the sediment and 510 OTUs from the foraminiferal dataset, of which 65 OTUs (TF) were assigned to phylum Retaria and all other 445 OTUs to their intracellular eukaryote content (TIFC). After the exclusion of Retaria OTUs from the sediment data, 1,571 OTUs (TS) remained for further analysis.

Rarefaction analysis indicates that the filtered OTU dataset reaches asymptote levels, allowing for richness comparison among samples for both the sediment and intracellular foraminiferal datasets (Supplementary Figure 3). One sample that exhibits the same OTU richness as the controls and is distant from the rest of sediment samples was discarded from the TS dataset (C1, Supplementary Figure 4). In TIFC dataset, most of the samples reached a satisfactory sequencing depth (7 samples above the upper quartile (127,312 reads per sample) and 14 samples above the median (90,673 reads per sample, Supplementary Figure 3B). Samples with less reads (e.g., M4C, M4D, M7A, M6B) had similar composition and grouped with the rest of the foraminiferal samples (see Figure 2, 4A), thus they were included in subsequent analysis.

### Identification of Foraminiferal Specimens and Phylogenetic Analysis of Foraminiferal OTUs

Taxonomic identification was based on the TF with the greatest number of reads in each specimen (87.22% ± 13.70% average foraminiferal reads across specimens; see last column of Table 1). Specimens M2E, M4D, and M7A could not be assigned to genus level, so their microscopic identification was adopted. All our specimens fall within the order Rotaliida. In Mokbaai, 11 specimens were identified as *Ammonia* sp., 10 as *Elphidium* sp. and 2 as *Haynesina* sp., whereas all 5 specimens of de Cocksdorp were identified as *Elphidium* sp. (Table 1).

**FIGURE 1:**
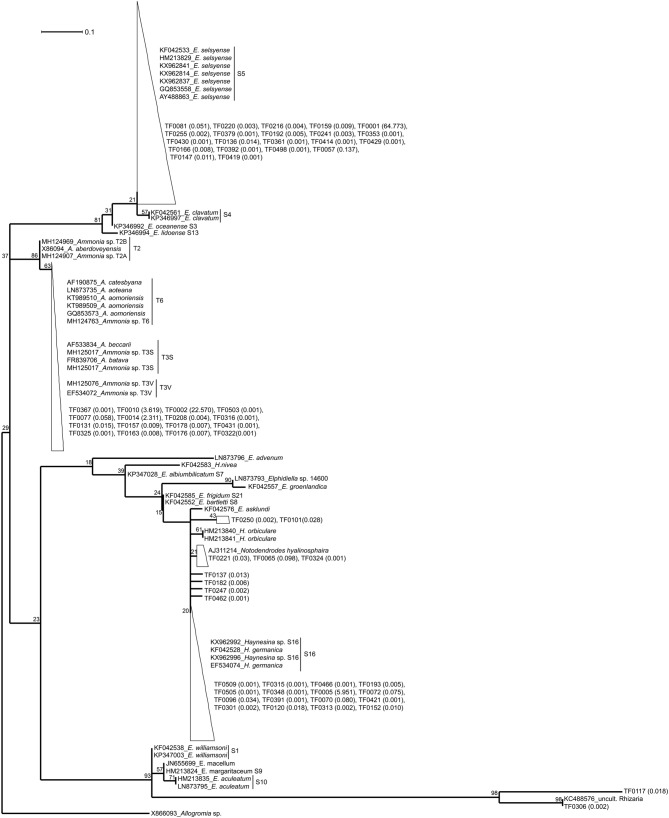
Maximum likelihood phylogenetic tree of the foraminiferal OTUs and their closest relatives. The tree was built based on partial SSU rDNA sequences (about 117 bp) and inferred using the ML method with the Kimura 2-parameter model. Collapsed branches are indicated by a triangle/polygon. The tree was rooted on *Allogromia* sp. (X86093). Bootstrap support values over 1000 replicates are shown at the nodes. The number in parenthesis following the TF sequences indicates their % relative abundance over the total number of foraminiferal sequences. The bar represents 0.1 average nucleotide substitutions per site.

For the maximum likelihood tree, representative TF sequences were aligned (ca. 117 bp; positions 1389–1510 of 18S rRNA gene) alongside 11 sequences of their closest relatives (97–100% similarity) and 37 sequences of known foraminiferal species. The majority of TF OTUs (21 TF, corresponding to 64.83% of all foraminiferal sequences) are similar (≥99% BLAST similarity) to *Elphidium* genetic type S5 and form a large clade (81% ML bootstrap support), including also genetic types S3, S4, and S13 (Figure 1). Another big cluster on the tree, with 86% bootstrap support, is that of *Ammonia* sp., comprising the genetic types T6, T3V, and T3S (*A. batava*). The second most abundant group of our sequences (16 TF; 24.99% of all foraminiferal sequences; 97-100% BLAST similarity to *Ammonia aomoriensis* (GQ853573) and >99% to *Ammonia* sp. T6 (KT989509)) falls within this cluster. Finally, there is a cluster of *Haynesina* sp.-related OTUs (25 TF), which is not a well-supported clade (only 20% bootstrap support). Among this cluster 16 TF (6.10% of all foraminiferal sequences) are highly similar (>98%) to *Haynesina* sp. S16 (KX962996, KX962992).

### Foraminiferal Intracellular Eukaryote Content Compared With Surrounding Sediment Eukaryote Communities

**FIGURE 2:**
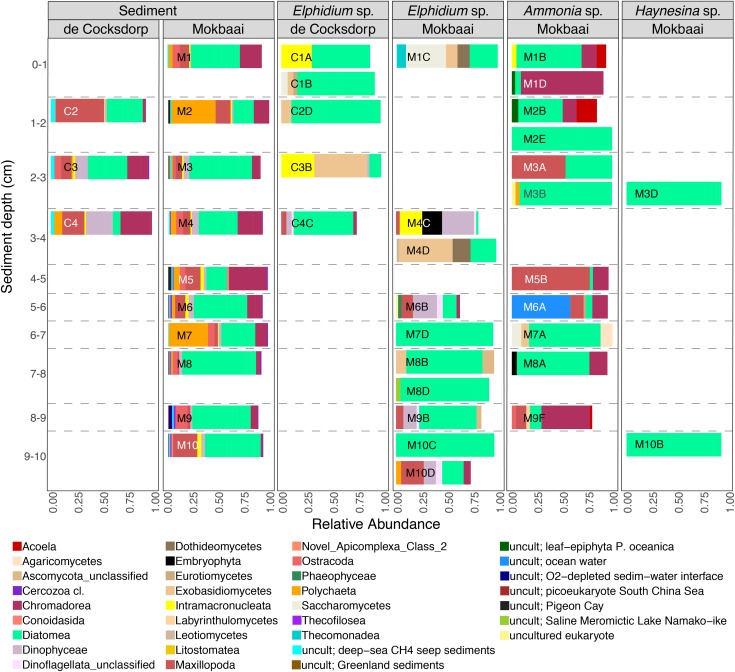
Relative abundance of eukaryote taxa at class level for foraminiferal intracellular eukaryote content (showing classes with > 2% abundance, i.e., 90.02% of all reads; foraminiferal OTUs excluded from the analyses) and communities of the surrounding sediments (showing classes with > 0.5% abundance, i.e., 83.20% of all reads). Foraminiferal species (*Ammonia* sp., *Elphidium* sp., *Haynesina* sp.) and sampling sites (de Cocksdorp, Mokbaai) are shown on the top grid. Taxa that are similar to uncultured eukaryotes are indicated by “uncult” followed by information on the environment of their closest relatives.

**FIGURE 3:**
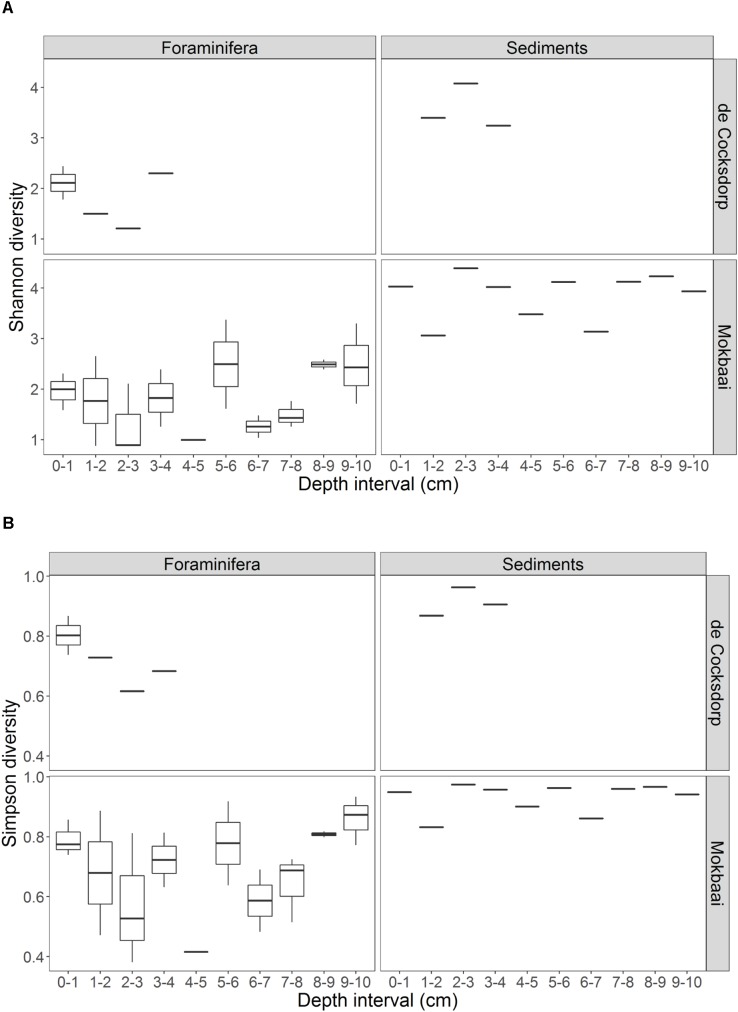
Summary of the alpha diversity, calculated by **(A)** Shannon and **(B)** Simpson indices, of foraminiferal intracellular content (excluding foraminiferal OTUs) and sediment communities. Foraminiferal communities were grouped per depth interval. There are multiple foraminiferal specimens for each depth interval (see Table 1, here shown by boxplots) but always one sediment sample per depth interval. Boxplots show the median (middle line) diversity; the lower and upper hinges correspond to the first and third quartiles (the 25th and 75th percentiles) of the diversity range; the upper and lower whiskers extend from the hinge to the largest and lowest value no further than 1.5 ^∗^ IQR from the hinge (where IQR is the inter-quartile range, or distance between the first and third quartiles).

TIFC reflected TS, but clear differences were observed in relative abundances (Figure 2). For example, diatoms (class Diatomea in Figure 2) were the most abundant eukaryotes in the majority of the foraminiferal specimens (51.36% relative abundance on average). They were also common in sediments, but generally at lower relative abundances (22.67% relative abundance on average). Alpha diversity measured using either the Shannon or Simpson index was significantly higher for TS than TIFC (ANOVA, *p* < 0.001, Figure 3).

The composition of TIFC appeared to be species-specific (Figure 2). The intracellular community of the two *Haynesina* sp. specimens consisted entirely of diatoms, and the same was true for two *Elphidium* sp. specimens (M7D, M10C). A variety of diatom genera was found in all three species (Supplementary Figure 5). Pennate genera, such as *Climacosphenia* sp. and *Petrodictyon* sp. were common in *Elphidium* sp. of surface sediments, whereas *Elphidium* sp. specimens from deeper sediments contained more *Thalassiosira* sp. and genera of the family Mediophyceae. Alongside diatoms, some *Elphidium* sp. specimens contained dinoflagellates (e.g., class Dinophyceae, 13–31% relative abundance in M4C, M6B, M9B, M10D), ciliates (class Intramacronucleata, 23–32% relative abundance in C1A, C3B, M4C) and fungal groups (e.g., class Saccharomycetes 39 % relative abundance in M1C, and class Exobasidiomycetes 51% in C3B and 52% in M4D). Metazoan classes were generally more abundant in *Ammonia* sp. specimens, i.e., Maxillopoda (relative abundance 10% in M9F to 76% in M5B; only 3–22% in some *Elphidium* sp. specimens), Nematoda (e.g., the class Chromadorea with 95% in M1D, 18% in M8A, 49% in M9F, but only 1–6% in *Elphidium* sp. specimens) and Acoela (e.g., 20% in M2B; none in *Elphidium* sp. specimens).

**FIGURE 4:**
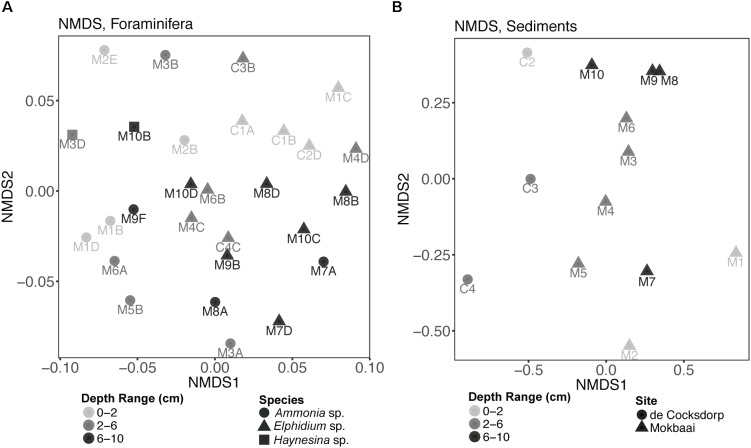
Non-metric multidimensional scaling (nMDS) plots of **(A)** foraminiferal intracellular eukaryote content (excluding foraminiferal OTUs) and **(B)** communities of the surrounding sediments. Samples from different sediment depths (cm) are grouped in three depth ranges: 0–2, 2–6, and 6–10 cm. “M” indicates foraminiferal specimens and sediment samples from Mokbaai; “C” indicates foraminiferal specimens and sediment samples from de Cocksdorp. nMDS was based on a Bray-Curtis distance and the stress for foraminifera was 0.2243, whereas for sediments 0.1280.

Non-metric multidimensional scaling (nMDS) analysis of TIFC (Figure 4A) showed that the three foraminiferal species are well separated in the ordination space, followed by separation based on the depth range from which the specimens derived. TIFC of *Ammonia* specimens generally clustered together, however three specimens (M2E, M3B, M2B) were separated from the rest and closer to *Elphidium* and *Haynesina* specimens. TIFC in these specimens was dominated by diatoms, as was the case with *Elphidium* and *Haynesina* specimens. Species was a significant factor (PERMANOVA, *F* = 2.884, *p* = 0.001) for the observed community variance, followed by sediment depth range (PERMANOVA, *F* = 1.447, *p* = 0.040). This was also true for the distribution of intracellular diatom genera (species: PERMANOVA, *F* = 2.030, *p* = 0.016; depth range: PERMANOVA, *F* = 1.530, *p* = 0.047). In contrast to overall TIFC community composition, which was driven by the depth range, pairwise comparisons carried out separately for each species within each depth range (0–2, 2–6, and 6–10 cm), indicated no significant differences among TIFC of the same depth range groups (pairwise MANOVA, *p* > 0.14 within and among species, with Benjamini–Hochberg adjustment). Additionally, the significance of site (de Cocksdorp vs. Mokbaai specimens) was evaluated, after excluding Mokbaai specimens from 5 cm and deeper, as no living specimens were found deeper than 4 cm depth in de Cocksdorp. The analysis showed that site was not a significant factor (PERMANOVA, *F* = 1.038, *p* = 0.401). In contrast to the foraminiferal intracellular eukaryote content, the sediment eukaryote community between Mokbaai and de Cocksdorp was different (Figure 4B). Site was the most significant factor in sediments (PERMANOVA, *F* = 3.658, *p* = 0.001), followed by depth range (PERMANOVA, *F* = 2.056, *p* = 0.009).

**FIGURE 5:**
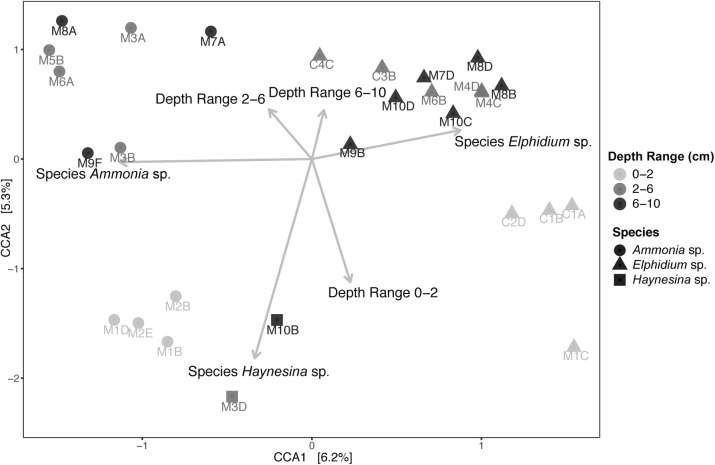
Canonical Correspondence Analysis (CCA) of foraminiferal intracellular eukaryote content (excluding foraminiferal OTUs) and potential explanatory variables. Specimens from different sediment depths (cm) are grouped in three depth ranges: 0–2, 2–6, and 6–10 cm. “M” indicates foraminiferal specimens from Mokbaai and “C” from de Cocksdorp. Arrows, indicating the correlation between the canonical axes and the explanatory variables, are only shown for the significant variables. Average organic carbon content (in weight % of dry sediment), average total nitrogen content (in weight % of dry sediment) and average C/N per depth range (C mol/ N mol) were also included in the CCA model but were not significant (*p* > 0.1). Organic carbon and nitrogen content values are shown in Supplementary Table 2.

Subsequently, Canonical Correspondence Analysis (CCA) was performed to account for the impact of various environmental factors on the observed foraminiferal intracellular eukaryote content variance (Figure 5). A total of 24.80% of the observed community variance was explained by the constraints (foraminiferal species, sediment depth range and the per-depth range average nitrogen (N) and organic carbon (C), as well as their ratio (C/N); see Supplementary Table 2 for C and N concentrations). Overall, our chosen CCA model was significant (ANOVA, *F* = 1.154, *p* = 0.03). Foraminiferal species was the main driving factor in explaining the foraminiferal intracellular eukaryote content (ANOVA, *F* = 1.421, *p* = 0.004), followed by sediment depth range (ANOVA, *F* = 1.160, *p* = 0.041). No other factor contributed significantly to the observed foraminiferal intracellular eukaryote content variance. A similar CCA model was built for the sediment communities (Supplementary Figure 6), which was overall significant (ANOVA, *F* = 1.867, *p* = 0.004) and confirmed that site was the most significant factor (ANOVA, *F* = 2.566, *p* = 0.001), followed by sediment depth range (ANOVA, *F* = 1.676, *p* = 0.004). All the other factors (including organic carbon and nitrogen contents) were not significant but contributed to the overall variance explained by the constraints of the model (48.28%).

## Discussion

### Metabarcoding of the 18S V9 Region: A Useful Tool for the Taxonomic Placement of Intertidal Foraminifera

Correct taxonomy is pivotal in understanding species-specific trophic behavior and benthic food-web structure. Based on this study, metabarcoding of the 18S V9 region and using PR^2^ (Guillou et al., 2013) as reference database allows determining the taxonomic placement of foraminiferal specimens. The taxonomy suggested by PR^2^ was confirmed by BLAST results (Table 1) and further supported by phylogenetic analysis (Figure 1). TF OTUs were assumed to derive from the specimens’ own DNA. We cannot preclude the possibility of foraminifera praying on other foraminifera (e.g., Lipps, 1983), however, on average 87% of the TF reads within our specimens were taxonomically assigned (and confirmed by phylogenetic analysis) to the same foraminiferal species as the species assigned based on morphology. Thus, in this case, foraminifera cannibalism is unlikely to play an important role. Morphological identification of some foraminiferal specimens is a difficult task and can lead to wrong taxonomic assignment. For example, similar morphologies have been documented for different *Ammonia* sp. genetic types, such as T1, T2, T6, and T10 (Hayward et al., 2004; Schweizer et al., 2011). The same is also true for Elphidiidae (e.g., Pawlowski and Holzmann, 2008; Darling et al., 2016), particularly in the case of small specimen sizes. Thus, the importance of integrating morphological and molecular results to secure identification and taxonomic placement of foraminiferal species has been recognized and established in recent benthic foraminiferal studies (e.g., Schweizer et al., 2008; Pillet et al., 2013; Darling et al., 2016; Roberts et al., 2016). However, care should be taken when assigning taxonomy at genus/species level, as results may differ depending on the database used. For example, based on our results, SILVA database tends to assign sequences of the order Rotaliida to *Ammonia* sp., although BLAST and phylogenetic analysis confirmed that many of our specimens belonged to *Elphidium* sp. or *Haynesina* sp. The PR^2^ database was superior in the assignment of our benthic foraminiferal sequences and it has also been curated to include all planktonic foraminiferal rDNA sequences (Morard et al., 2015, 2018). We therefore recommend the use of the PR^2^ database for the assignment of rotaliid foraminifera at genus level, yet we stress the importance of following up with phylogenetic analysis for secure identification. Nonetheless, care should be taken as the V9 region is a very small region of the 18S rRNA gene. In this case, the alignment length was only about 117 nucleotide sites, which, in addition to the genetic variability within elphidiids, constrains the robustness of our phylogenetic analysis. The observed low bootsrap support values make the phylogenetic relationships difficult to intrepret, and, hence the phylogentetic tree here serves only as as a visualization tool for within-clade sequence similarity. Comparison of our sequences to databases is sufficient for a secure taxonomic assignment (similarities ≥ 97%).

Phylogenetic analysis confirms that our specimens are part of the order Rotaliida, belonging to Elphidiidae, Rotaliidae, and Nonionidae families (Holzmann and Pawlowski, 2017). The large *Elphidium*-related clade on our tree (81% ML bootstrap support, Figure 1) is matching clade F of the phylogenies presented in Pillet et al. (2013) and Darling et al. (2016). The morphologically similar but distinct genetic types S4 and S5 is a good example of the taxonomic confusion within elphidiids (Roberts et al., 2016), as genetic type S4 has been considered as part or subspecies of *E. excavatum*, till the latest suggestion by Darling et al. (2016) to assign the name *E. clavatum* to the genetic type S4 and the name *E. selseyense* to genetic type S5. *Elphidium* sp. genetic type S5 has been found before in the Mokbaai mudflat (Schweizer et al., 2011; Jauffrais et al., 2018) and in other mudflats in the United Kingdom (Schweizer et al., 2011; Darling et al., 2016) and France (Ertan et al., 2004), and there have also been occurrences in the Baltic Sea (Schweizer et al., 2011). It seems to be a rather widespread intertidal taxon, tolerant to relatively large variations of temperature and salinity (Darling et al., 2016). The rest of the *Elphidium* sp. in our phylogeny form separate clades, which indicates a paraphyletic group and is in agreement with previous phylogenetic placements (Schweizer et al., 2011; Pillet et al., 2013; Darling et al., 2016). For example, clade A of Pillet et al. (2013) and Darling et al. (2016) with *E. williamsoni* (genetic type S1), *E. macellum* [Patagonia branch on Darling et al. (2016)], *E. margaritaceum* 1 (genetic type S9) and *E. aculeatum* (genetic type S10), is a separate branch on our phylogeny, which clusters together with a Rhizaria sequence retrieved from the waters of the Scotian Shelf (Dasilva et al., 2014).

We only had two *Haynesina* sp. specimens, both retrieved from the Mokbaai mudflat. All the *Haynesina*-related OTUs were similar (>98% BLAST similarity) to genetic type S16 (*Haynesina germanica*), forming part of clade C (Pillet et al., 2013; Darling et al., 2016). However, the bootstrap support for this clade on our tree is extremely low (20%, Figure 1). This group of sequences is branching with *H.orbiculare*, which alongside S16 is part of clade C in Pillet et al. (2013) and Darling et al. (2016). In addition, *E. asklundi* appears on this branch in our phylogeny, whereas it is part of the sister clade D in the aforementioned studies. *Haynesina* sp. S16 has been retrieved from sediments in Den Oever and Texel, Netherlands (Schweizer et al., 2008), and it has a similar geographic distribution to that of *Elphidium* sp. S5 (Darling et al., 2016).

*Ammonia* sp. sequences form a separate clade (86% ML support, Figure 1) on our phylogenetic tree, consisting of two branches. The first branch is that of *Ammonia* genetic types T2A (*A. aberdoveyensis*) and T2B, recently suggested as subgroups of T2, based on both SSU and LSU (large subunit) rDNA, by Bird et al. (2019). The second one is that of genetic types T6 (often called *A. aomoriensis*), T3S (*A. batava)* and T3V. Our *Ammonia* sequences were similar (>97%) to a specimen from the Kiel Fjord (SW Baltic Sea), identified as *A. aomoriensis* (GQ853573). The second *Ammonia* branch (63% ML support, Figure 1) on our phylogenetic tree is in agreement with the results based on partial SSU and LSU sequences (Schweizer et al., 2011), where *Ammonia* sp. specimens from the Kiel Fjord cluster with the genetic type T6. This cosmopolitan genetic type has been found across different geographic areas, e.g., in the North Sea (Langer and Leppig, 2000), in the sediments of brackish waters of Japan (Nomura and Seto, 1992, Nomura, 2003; Takata et al., 2006) and in the Yellow Sea of China (Xiang et al., 2008). The figure holotype of T6 from Honshu, Japan was named *A. aomoriensis* (Asano, 1951) but its adoption for genetic type T6 is under debate (Hayward et al., 2004; Bird et al., 2019).

In previous studies the number of nucleotide sites used in phylogenies was considerably larger [1686 sites in Pillet et al. (2013) and 601 sites in Darling et al. (2016)] than ours (117 sites), therefore producing statisticaly more robust topologies. However, there is generally a good agreement between published tree topologies [Figure 1 in Pillet et al. (2013), Figure 2 in Darling et al. (2016)] and ours (Figure 1), with the placement of representative genetic types in the same clades A-F (except members of clades E and D that cluster in sister branches rather than in the same one on our tree). Even though a thourough phylogenetic placement of the various elphidiid genetic types is outside the scope of this study, our results are consistent with the established clades of the aformentioned studies. Notably, clade F in our analysis branches separately from clades A and B-E, which matches better the second scenario presented in Pillet et al. (2013). According to this scenario, rooting is done on *Ammonia* sp. and clade F branches separately from the rest of the clades, suggesting a closer evolutionary relationship between elphidiids and nonioiniids.

Our phylogenetic analysis corroborates the BLAST and PR^2^ results for the assignment of the genetic types *Elphidium* sp. S5, *Haynesina* sp. S16, and *Ammonia* sp. T6 to our specimens, which is consistent with the biogeographic distribution of these genetic types. Moreover, the molecular identification is supported by SEM observations (see Figure 2 for *Haynesina* sp. S16 and Figure 5 for *Elphidium* sp. S5 in Jauffrais et al., 2018; Figure 1 for *Ammonia* sp. T6 in Koho et al., 2018) of specimens sampled from the same sites, as these match the morphological characteristics of the above genetic types.

### Trophic Preferences of Intertidal Foraminifera

Here, for the first time, we used a metabarcoding approach to investigate *in situ* feeding patterns of intertidal benthic foraminifera. This method, although with some known pitfalls related to amplification biases (e.g., Logares et al., 2014; Pawluczyk et al., 2015), is known to perform better compared to conventional amplicon sequencing, as it allows an in-depth community investigation. Our results successfully show the distinct food preferences of different foraminiferal species despite them inhabiting the same benthic environment. If foraminifera were randomly deposit feeding on sediments and ambient eukaryotes, their intracellular eukaryote communities would be expected to be (i) similar between species and (ii) a close reflection of the sediment composition. This was not the case as the constrained multivariate analysis (Figure 5) indicates that foraminiferal species is the driving factor in shaping TIFC. In addition, whilst the sediment community was significantly different at the two study locations (Supplementary Figure 6), the TIFC was not affected by site. Furthermore, the greater alpha diversity of the TS compared to TIFC (Figure 3) suggests that foraminifera may have some preferences with regards to what taxa they feed on from their environment and therefore do not simply reflect the biota in the surrounding sediments. This diversity, however, can only be regarded as a proxy of potential trophic preferences and not as solid evidence, as the difference in sample material (1–1.5 g sediment vs. a single foraminiferal cell) could have an effect on the observed alpha diversity.

In most of our *Ammonia* sp. specimens, targeting the 18S V9 region revealed an enrichment of metazoan classes (e.g., Acoela, Chromadorea, Maxillopoda), implying that in addition to feeding on phototrophs (e.g., diatoms), *Ammonia* sp. has a tendency toward active predatory behavior. Indeed, *Ammonia tepida* has been shown in laboratory experiments to actively entrap nematodes with its pseudopodial network and empty the nematode’s soft tissue within 18 h of initial contact (Dupuy et al., 2010). In addition, a few other benthic foraminifera have been shown to feed on metazoans (Langer and Bell, 1995; Goldstein, 1999; Suhr et al., 2008). Until now, however, *in situ* evidence of this behavior is lacking. Further *in situ* observations on different benthic foraminiferal species are needed to elucidate their carnivorous behavior in different environmental conditions and to fully understand the position of foraminifera in the benthic food web.

The intracellular eukaryote communities of our *Elphidium* sp. and *Haynesina* sp. specimens were mainly dominated by diatoms. Foraminiferal ingestion of diatoms has been documented in numerous feeding experiments (e.g., Larkin et al., 2014; Jeffreys et al., 2015; LeKieffre et al., 2017). In addition, Austin et al. (2005) observed that *H. germanica* specimens were drawing the provided diatoms toward their aperture with their pseudopodia and SEM images indicated a characteristic cracking pattern of the diatom frustules. In another laboratory experiment where *H. germanica* was provided with diatoms and sewage-derived particulate organic matter, a fourfold increase was observed after 2 weeks in the levels of diatom fatty acid biomarker inside the foraminifera (Ward et al., 2003). In a field study of Schönfeld and Numberger (2007), an increase in the populations of *Elphidium excavatum clavatum* was found to occur simultaneously with the phytodetritus deposition. The authors suggested that *Elphidium e. clavatum* ingests fresh diatoms immediately upon deposition from the water column and does not wait for incorporation of the organic detritus into the sediment. Our results support these previous observations, and imply a predominantly planktivorous feeding mode for *Elphidium* sp. and *Haynesina* sp.

In addition to feeding, the acquisition of phototrophs by benthic foraminifera may be linked to photosymbionts or the phenomenon of kleptoplasty, i.e., the assimilation and maintenance of foreign chloroplasts. Both elphidiids and some nonioniids (e.g., *Haynesina and Nonionellina*) have the capacity to retain chloroplasts from algal prey (e.g., Lopez, 1979; Cedhagen, 1991; Pillet et al., 2011; Jauffrais et al., 2018). The active role of kleptoplasts in inorganic carbon assimilation by *H. germanica* was recently demonstrated by a paired TEM-NanoSIMS observations in light conditions, suggesting a functional photosynthetic role of kleptoplasts in *H. germanica* (LeKieffre et al., 2018). In the same study, moderately ^15^N-labeled kleptoplasts were observed in both light and darkness, which might indicate their involvement in nitrogen assimilation. Kleptoplasts may be involved in carbon and nitrogen uptake in other intertidal kleptoplast-bearing foraminiferal species as well, however, further analyses are needed to confirm their function. Molecular analysis of the kleptoplasts of *Haynesina* sp. and *Elphidium* sp., have indicated that kleptoplasts in these foraminifera originate exclusively from diatoms, however, there appears to be no clear specificity for diatom type (Pillet et al., 2011). In photosymbiont-bearing foraminifera *Pararotalia calcariformata*, the presence of 17 different endosymbiontic diatoms has been recently linked to symbiont shuffling as an adaptation strategy under thermal stress (Schmidt et al., 2018). Our data confirms that *Elphidium* sp. and *Haynesina* sp. contain a wide range of diatoms (Supplementary Figure 5), thus implying that the kleptoplasts may have originated from a variety of diatom species. In addition, our data shows that the foraminiferal intracellular diatom community changes with sediment depth. As photosynthesis is restricted to surface sediments, where light is readily available, our observations suggest that in the surface, pennate diatoms found inside *Elphidium* sp. specimens may be linked to kleptoplasty, and diatoms found in specimens from deeper sediments (e.g., *Thalassiosira* sp.) may be taken up predominantly as a food source. However, 16S rRNA gene metabarcoding of more specimens is needed to confirm diatom specificity patterns in the intracellular foraminiferal communities.

The intracellular eukaryotic community of some of our *Elphidium* sp. specimens also contained a high relative abundance of dinoflagellates and ciliates. In the feeding study of Lee et al. (1966), various species of littoral foraminifera, including *Elphidium* sp., were introduced to multiple carbon sources, including dinoflagellates. No dinoflagellates were ingested, and hence authors concluded that littoral foraminifera only fed on selected species of diatoms, chlorophytes and bacteria. Similarly, Duffield et al. (2014) observed a lack of positive response of foraminifera to net hauls dominated by dinoflagellates, except in the case of the species *Leptohalysis catella*, which increased in abundance when dinoflagellates were provided as a food source. Alternatively to being a food source, dinoflagellate DNA occurrence in our specimens may be related to a symbiotic relationship. Symbiosis between the dinoflagellates and planktonic foraminifera is well-known (Gast and Caron, 1996; Garcia-Cuetos et al., 2005; Pochon and Gates, 2010; Siano et al., 2010) but for benthic foraminifera only reported for large miliolids (Pawlowski et al., 2001).

In some of our specimens (particularly in *Elphidium* sp. C3B and M4D), there was high relative abundance of fungal DNA. The presence of fungal fruiting bodies of Ascomycetes has been observed before (Kohlmeyer, 1984, 1985) and it was suggested that the foraminiferal test chambers can serve as a protective niche for thin-walled fungal fruiting bodies (Kohlmeyer and Volkmann-Kohlmeyer, 1989) or that the protein-rich organic lining of the foraminiferal cell serves as nutrient source for the developing fungal ascocarps (Kohlmeyer, 1984). In our case, we cannot be certain of the presence of active fungal parts within our specimens based on the presence of fungal DNA alone. It is also possible that foraminifera acquired some fungal DNA attached onto sediment and diatom frustules while feeding.

The depth range, in which the specimens were found, was another significant factor for the observed intracellular eukaryote community variance inside our foraminiferal specimens (Figure 5). This makes sense, as sediment depth was also a significant factor for the community variance in the sediments, meaning that different eukaryotes are found at different sediment depths. Thus, foraminiferal specimens living at different sediment depths would have access to different eukaryote communities. The depth distribution of intertidal foraminifera in the sediment is typically focused on top sediments (e.g., Langezaal et al., 2003; Thibault De Chanvalon et al., 2015), yet intertidal foraminifera have been reported to occupy relatively irregular in-sediment distributions with living specimens occurring at tens of centimeters depth (Moodley and Hess, 1992). However, the activity of *Ammonia* sp. has been suggested to decline and even enter a state of dormancy in low-oxygen conditions (Maire et al., 2016; LeKieffre et al., 2017; Koho et al., 2018) that typically prevail in deeper sediments. Based on our study, it is likely that some of the specimens living in deeper sediment horizons were still actively grazing in oxygenated microenvironments, for example close to macrofaunal burrows. The sediments, especially at the Mokbaai site, were heavily bioturbated, which has been shown to be instrumental to the vertical distribution of intertidal foraminifera (Bouchet et al., 2009; Maire et al., 2016).

The general mechanisms of competition and adaptation in different environmental conditions can generate and enhance the phenomenon of niche partitioning, which has been documented among foraminifera (e.g., Aurahs et al., 2009; Weiner et al., 2012). Benthic foraminifera are known to adapt to a variety of habitats and this ability may be related and enhanced by their species-specific trophic preferences. It has been suggested that different feeding preferences among species could be an advantage in an environment where competition for space and food is high (Enge et al., 2014). This would be particularly true in areas of high cell densities, which can be the case in intertidal microhabitats (e.g., Murray, 2006; Tsuchiya et al., 2018). Moreover, intertidal zones are dynamic areas where environmental conditions change rapidly, thus creating unique microhabitats. Therefore, a varied and species-specific trophic behavior, as suggested by our results, can be an advantage in such rapidly changing environments. However, future studies with more specimens are required to clarify the potential species-specific diet preferences of benthic foraminifera.

## Conclusion

To the best of our knowledge, this is the first study to use metabarcoding of the small subunit ribosomal DNA (SSU rDNA) with a view to gaining insights into the trophic preferences of intertidal foraminifera and their role in the benthic food web. In terms of their trophic behavior, benthic foraminifera are likely to have species-specific preferences. *Ammonia* sp. showed a tendency toward being a secondary consumer and possibly preying actively on small eukaryote classes, such as Acoela, Nematoda, and Maxillopoda. Elphidiids and nonioniids (*Elphidium* sp., *Haynesina* sp.) showed a more herbivorous tendency with a clear preference for phototrophs, which could be related to kleptoplasty. Moreover, our results suggest that the V9 region of the 18S rRNA gene can be used for secure taxonomic assignment and phylogenetic placement of foraminifera. Metabarcoding of the 18S V9 region allowed us to confidently identify our specimens and assign their genetic types (*Elphidium* sp. S5, *Haynesina* sp. S16, and *Ammonia* sp. T6).

## Author Contributions

P-MC carried out amplifications for MiSeq library preparations, bioinformatics, and statistical analysis. IS extracted the DNA and carried out initial tests with the primers. IS and KK designed and carried out sampling and processing of samples in the field. KK conceived the study and did the carbon and total nitrogen analysis. CB assisted with the protocol for foraminiferal DNA extractions and with phylogenetic analysis, and did the genotyping. G-JR assisted with sampling co-ordination. P-MC, IS, and KK contributed to interpretation of results and P-MC drafted the manuscript. All authors contributed to the final version of the manuscript.

## Conflict of Interest Statement

The authors declare that the research was conducted in the absence of any commercial or financial relationships that could be construed as a potential conflict of interest.
