# Supplementary material for: Metabarcoding Insights Into the Trophic Behavior and Identity of Intertidal Benthic Foraminifera
Source: Front Microbiol. 2019 May 28;10:1169. doi: 10.3389/fmicb.2019.01169 (PMC6547873; doi:10.3389/fmicb.2019.01169)
Supplement: Supplementary file 1 [file Data_Sheet_1.PDF]

Supplementary Material

1 Supplementary Tables

Supplementary Table 1. Number of living foraminiferal specimens retrieved per site, genus and depth range.

Site	Genus	No. of specimens per depth range			Total
		0-2 cm	2-6 cm	6-10 cm	
Mokbaai	*Ammonia* sp.	4	4	3	23
	Elphidium sp.	1	3	6	
	Haynesina sp.	0	1	1	
de Cocksdorp	*Ammonia* sp.	0	0	0	5
	Elphidium sp.	3	2	0	
	Haynesina sp.	0	0	0	
	Sum	8	10	10	28

Supplementary Table 2. Sediment contents in organic carbon, nitrogen, their ratio (C/N) and their averages for the selected depth ranges.

Site	Depth (cm)	N	C	C/N_wt	C/N_mol	Depth Range (cm)	N Average	C Average	C/N_wt Average	C/N_mol Average
M	0-1	0.06	0.63	10.77	12.57	0-2	0.07	0.75	10.25	11.96
M	1-2	0.09	0.87	9.73	11.35					
M	2-3	0.11	1.03	9.01	10.52	2-6	0.07	0.74	10.47	12.21
M	3-4	0.07	0.70	10.28	12.00					
M	4-5	0.06	0.64	10.99	12.82					
M	5-6	0.05	0.60	11.58	13.51					
M	6-7	0.05	0.55	12.01	14.02	6-10	0.05	0.63	11.54	13.47
M	7-8	0.06	0.68	11.33	13.22					
M	8-9	0.05	0.60	11.88	13.86					
M	9-10	0.06	0.69	10.95	12.78					
C	0-1	0.14	1.10	8.10	9.45	0-2	0.13	1.08	8.26	9.63
C	1-2	0.13	1.06	8.41	9.81					
C	2-3	0.22	1.54	6.89	8.04	2-6	0.15	1.18	8.31	9.69
C	3-4	0.08	0.81	9.73	11.35					

N = total nitrogen content (weight % of dry sediment); C = organic carbon content (weight % of dry sediment); C/N_wt = C/N ratio (g% C/g% N); C/N_mol = C mol/ N mol.
M = Mokbaai; C = de Cocksdorp.

2 Supplementary Figures

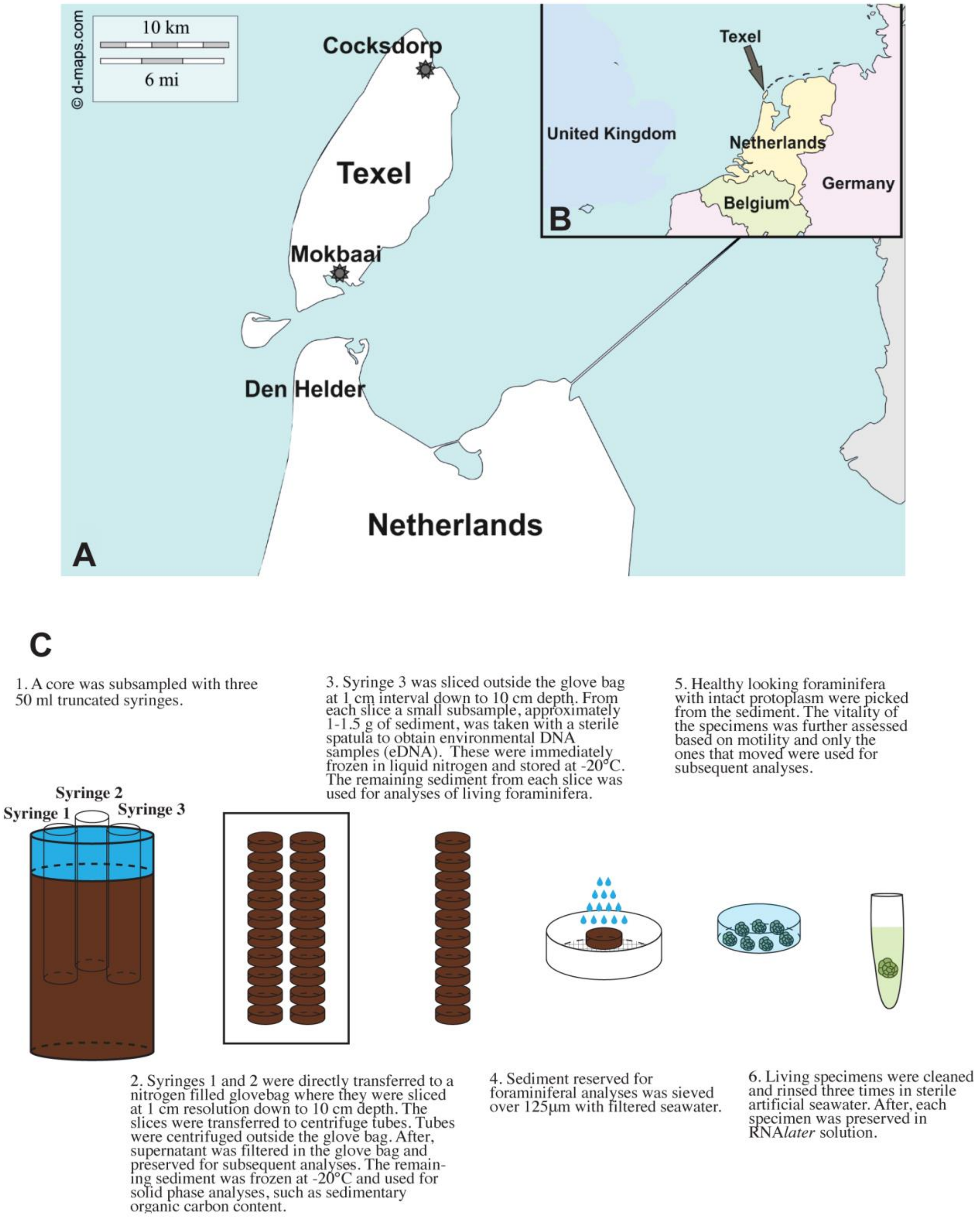

Supplementary Figure 1. Map of the sampling area. **(A)** Overview of the wider North Sea area. **(B)** The sampling sites (de Cocksdorp, Mokbaai) on the island of Texel, Netherlands. Map modified from http://d-maps.com/. **(C)** Schematic of the sediment and foraminifera sampling process.

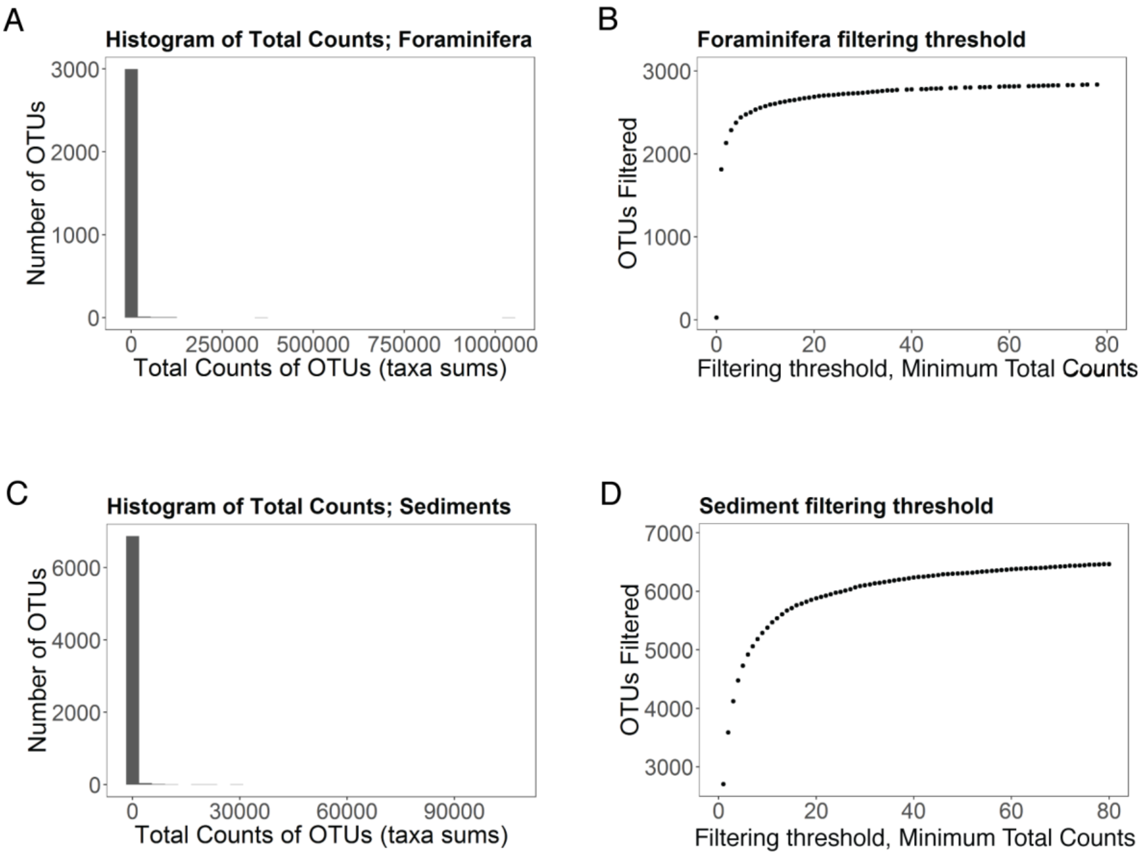

Supplementary Figure 2. Total counts (total number of observations of an OTU across all samples) in **(A)** the foraminiferal **(C)** the sediment dataset, and cumulative sum of OTUs that would be filtered against the total counts for **(B)** foraminiferal and **(D)** sediment datasets. A filtering threshold of ≥8 total counts was applied to foraminifera (retaining 99.86% of total sequence reads) and ≥10 total counts to sediments (retaining 99.03% of total sequence reads).

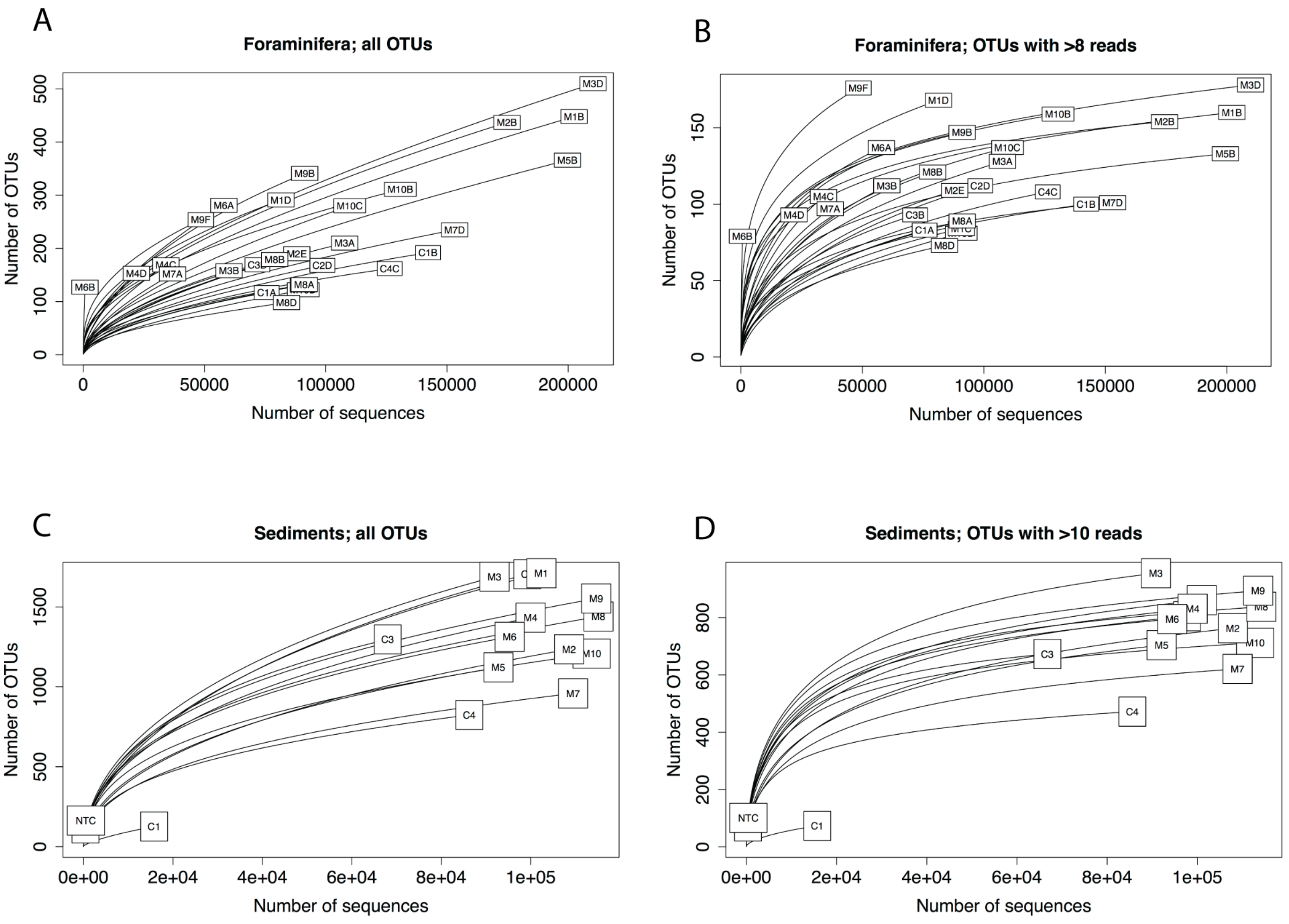

Supplementary Figure 3. Rarefaction curves of foraminiferal **(A, B)** and sediments datasets **(C, D)** using all the OTUs **(A, C)** and after application of the filtering threshold for OTUs containing low number of reads **(B, D)**.

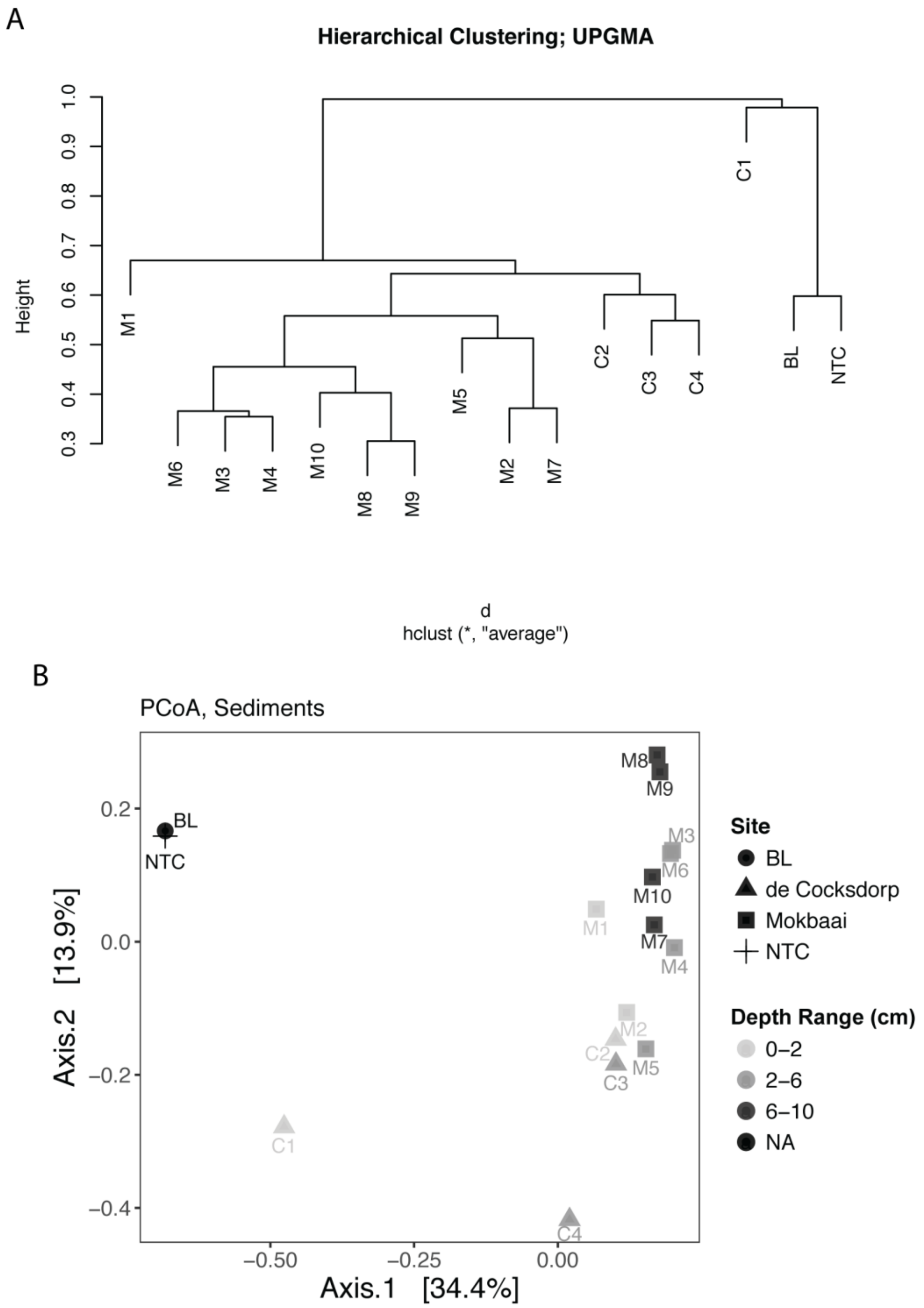

Supplementary Figure 4. (A) Hierarchical clustering of the sediment OTUs using the Unweighted Pair Group Method with Arithmetic Mean (UPGMA), based on a Bray-Curtis distance matrix. **(B)** Principal Coordinate Analysis (PCoA) of the sediment data. Samples are grouped by depth range (cm). BL=blank sample of the DNA extraction kit; NTC=PCR non-template control; NA=not applicable. “C” indicates de Cocksdorp and “M” Mokbaai samples.

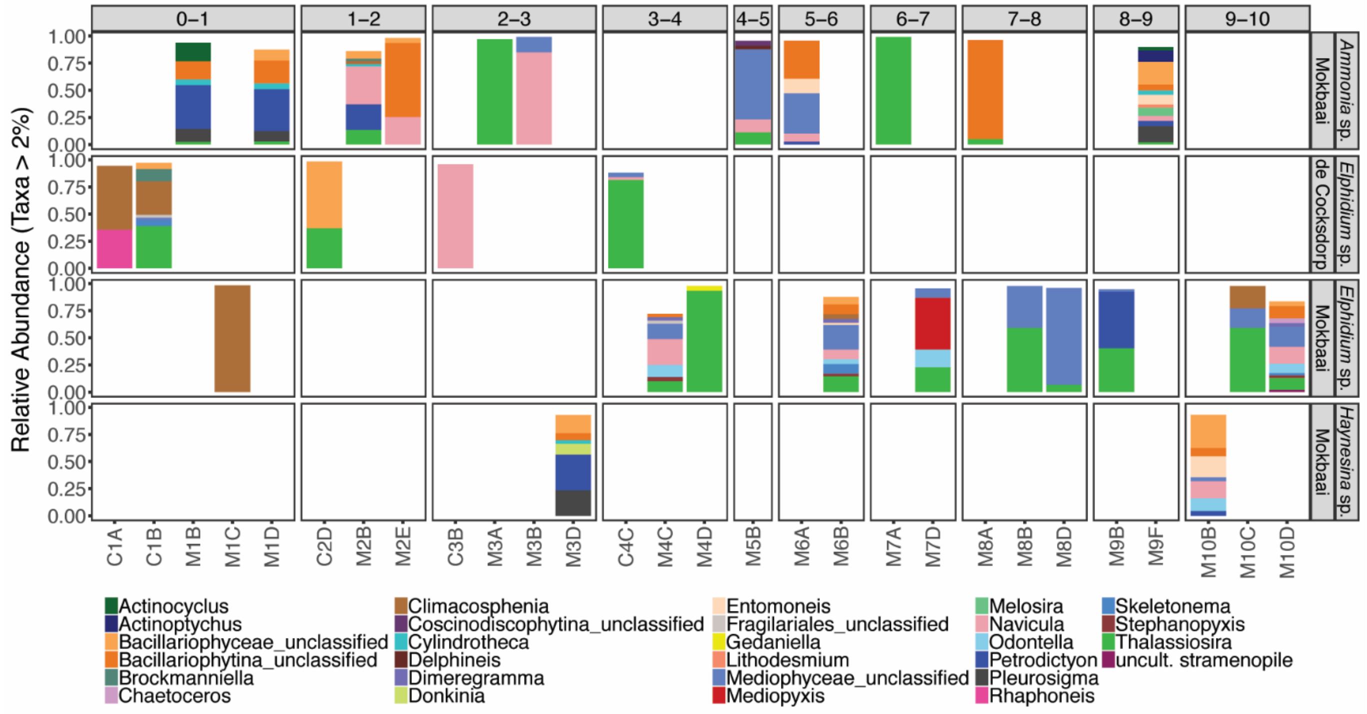

Supplementary Figure 5. Relative abundance of diatoms at genus level inside the foraminifera specimens (showing diatom genera with >2% abundance., i.e. 93.54% of all diatom sequences). Sediment depth intervals (cm) are shown on the top grid, foraminiferal species (*Ammonia* sp., *Elphidium* sp., *Haynesina* sp.) and sampling sites (de Cocksdorp, Mokbaai) on the side.

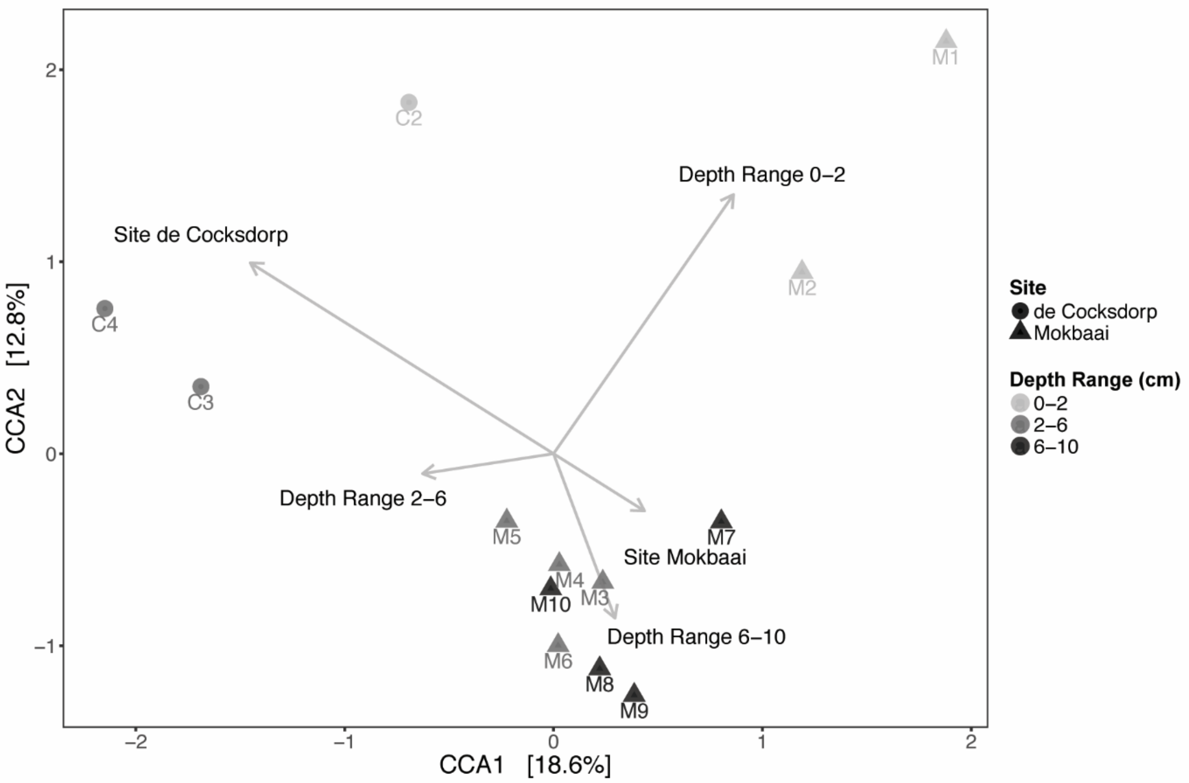

Supplementary Figure 6. Canonical Correspondence Analysis (CCA) of sediment communities. Samples from different sediment depths (cm) are grouped in three depth ranges: 0-2 cm, 2-6 cm and 6-10 cm. “C” indicates samples from de Cocksdorp and “M” from Mokbaai. Arrows, indicating the correlation between the canonical axes and the explanatory variables, are only shown for the significant variables. Average organic carbon content (in weight % of dry sediment), average total nitrogen content (in weight % of dry sediment) and average C/N per depth range (C mol/ N mol) were also included in the CCA model but were not significant ($p>0.1$).
